# Supplementary material for: KAP1 negatively regulates RNA polymerase II elongation kinetics to activate signal-induced transcription
Source: Nat Commun. 2024 Jul 12;15:5859. doi: 10.1038/s41467-024-49905-7 (PMC11245487; doi:10.1038/s41467-024-49905-7)
Supplement: Supplementary file 1 — Supplementary Information [file 41467_2024_49905_MOESM1_ESM.pdf]

Supplementary Information

KAP1 negatively regulates RNA polymerase II elongation kinetics to activate signal-induced transcription

Usman Hyder[1], Ashwini Challa[1], Micah Thornton[2], Tulip Nandu[2], W. Lee Kraus[2], and Iván D'Orso[1]*

[1]Department of Microbiology, The University of Texas Southwestern Medical Center, Dallas, TX 75390, USA

[2]Laboratory of Signaling and Gene Regulation, Cecil H. and Ida Green Center for Reproductive Biology Sciences, The University of Texas Southwestern Medical Center, Dallas, TX 75390, USA

*Corresponding author: Ivan.Dorso@utsouthwestern.edu

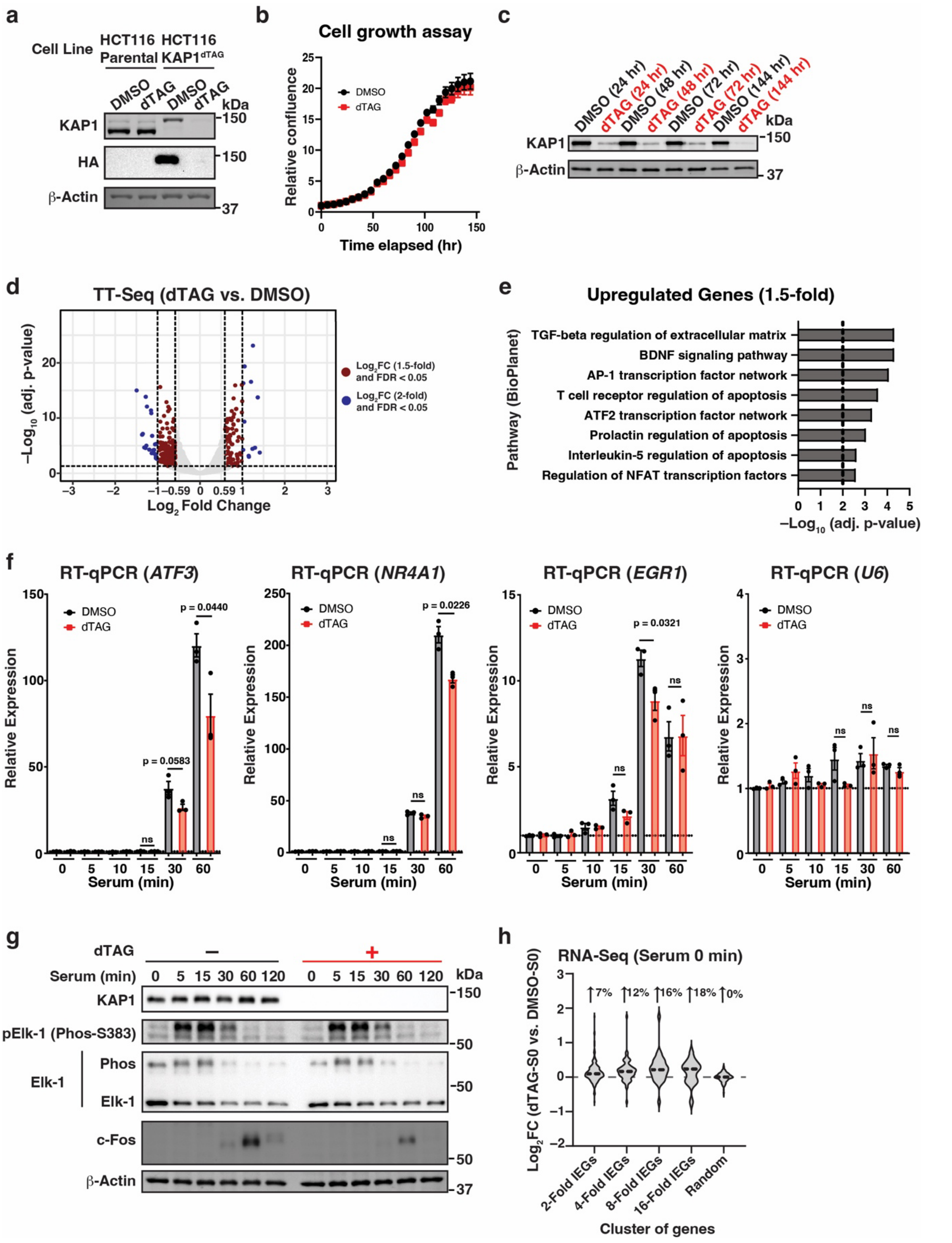

Supplementary Figure 1. Acute KAP1 depletion leads to virtually no transcriptional changes in homeostatic conditions. a dTAG characterization in HCT116:KAP1^{dTAG} and

HCT116 parental cells. Blots are representative of two independent experiments. **b** Cell growth assay showing relative cell confluence during a dTAG treatment time course. Data represents mean ± SEM (n=4). Black: DMSO, red: dTAG. **c** Western blots to monitor KAP1 degradation kinetics up to 6 days of dTAG treatment. Blots are representative of two independent experiments. **d** TT-Seq volcano plot (n=3 biological replicates, two-sided Likelihood Ratio Test using the Benjamini-Hochberg (BH) multiple comparisons adjustment, false discovery rate [FDR] < 0.05). Dots are colored based on Log_2FC values between DMSO- and dTAG- treatment according to the legend in the figure. **e** Pathway analysis for 1.5-fold upregulated genes identified from TT-Seq data. **f** RT-qPCR assay highlighting gene expression of three representative IEGs (*ATF3, NR4A1, and EGR1*) and one control gene (*U6*) in DMSO- and dTAG-treated cells during a serum stimulation time course. Data represents mean ± SEM (n=3 biological replicates, two-sided Student's t-test comparing DMSO to dTAG at each of the indicated time points). P values are indicated. Black: DMSO, red: dTAG. **g** Western blots showing induction of phosphorylated Elk-1 upon serum stimulation time course with and without dTAG treatment. Blots are representative of two independent experiments. **h** Violin plot showing changes in IEGs expression between DMSO and dTAG treated cells before serum stimulation (serum 0 min) organized by induction of expression: 2-Fold IEGs (n=236), 4-Fold IEGs (n=69), 8-Fold IEGs (n=34), and 16-Fold IEGs (n=16). Random (n=236) denotes random genes selected from non-DE genes (n=10,361). Data represents the Log_2FC value computed from RNA-Seq analysis between the DMSO-S0 and dTAG-S0 condition (generated from 3 biological replicates). The n number represents the number of genes in each cluster plotted. Median % changes in expression are listed above the violin in each IEGs cluster and Random non-DE genes. The median is also denoted as a dashed black line. Source data are provided as a Source Data file.

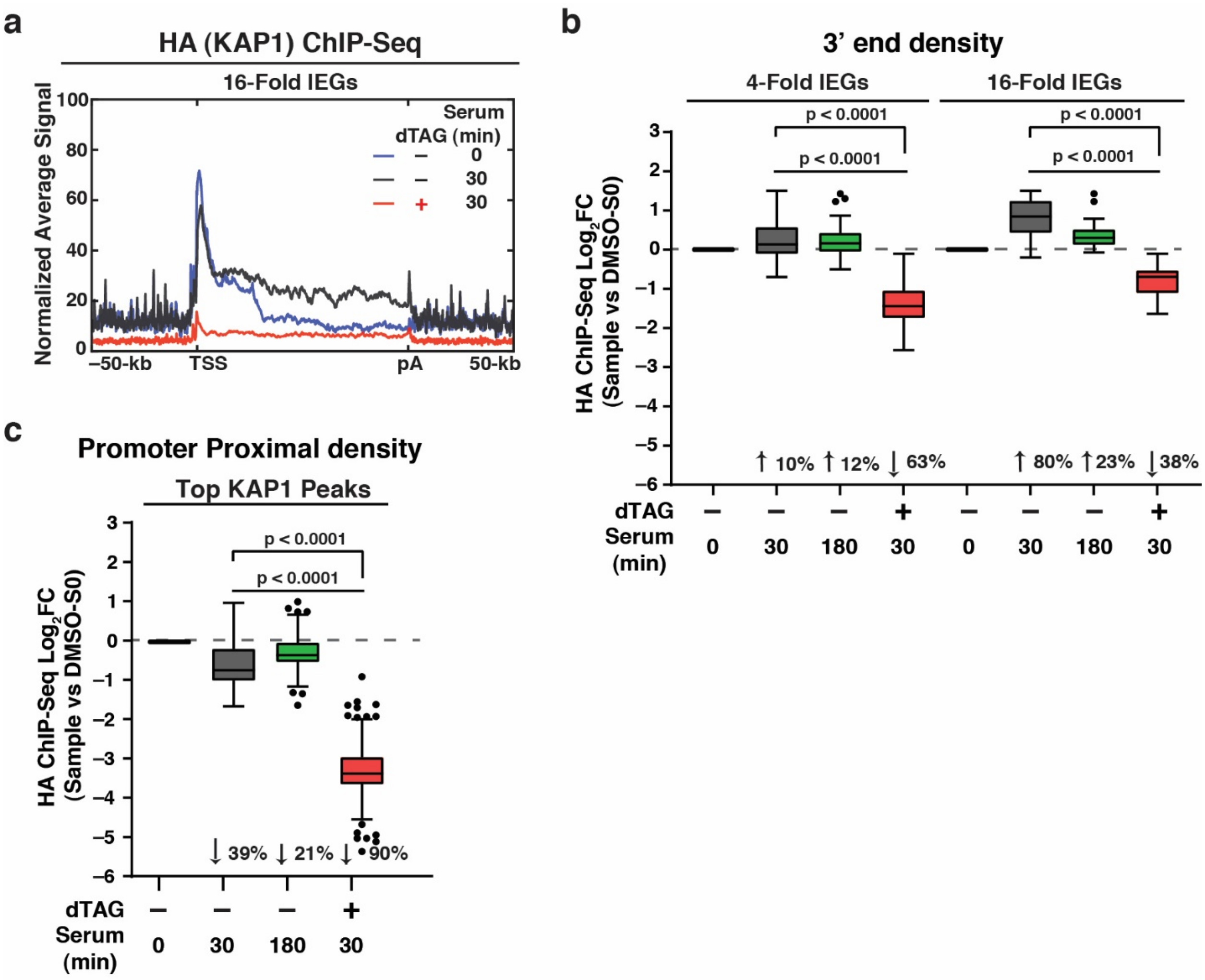

Supplementary Figure 2. KAP1 localizes to the gene bodies and 3' ends of IEGs upon serum stimulation. a HA (KAP1) ChIP-Seq metagene analysis at 16-Fold IEGs in the three indicated conditions (see legend for sample identification) with an extended X-axis to show 50-kb upstream the TSS and downstream the pA site. **b** HA ChIP-Seq quantitation's of KAP1 density at 4-Fold IEGs (n=69) and 16-Fold IEGs (n=16) at 3' ends of IEGs. Data represents the Log_2FC value for the respective sample (see X-axis) normalized to serum 0 min in DMSO-treated cells using ChIP-Seq signal from 2 biological replicates. The n number represents the number of genes in each cluster plotted. The Tukey plots indicate the median (black center line), the first and third quartiles (edges of the box) and 1.5 × interquartile range below and above the box as whiskers. Dots are presented as genes with normalized signal beyond these defined ranges. Statistics were calculated between the dTAG plus serum treatment condition and the respective condition shown on the Tukey plot. Two-sided Wilcoxon signed-rank test. P values are indicated. **c** HA ChIP-Seq quantitation's of KAP1 density at the top KAP1 peaks at PP regions (n=181, 19 genes were removed from the top 200 peaks because they contained no signal in at least one condition). Data represents the Log_2FC value for the respective sample (see X-axis) normalized to serum 0 min in DMSO-treated cells using ChIP-Seq signal from 2 biological replicates. The n number represents the number of genes in each cluster

plotted. The Tukey plots indicate the median (black center line), the first and third quartiles (edges of the box) and 1.5 × interquartile range below and above the box as whiskers. Dots are presented as genes with normalized signal beyond these defined ranges. Statistics were calculated between the dTAG plus serum treatment condition and the respective condition shown on the Tukey plot. Two-sided Wilcoxon signed-rank test. P values are indicated. Source data are provided as a Source Data file.

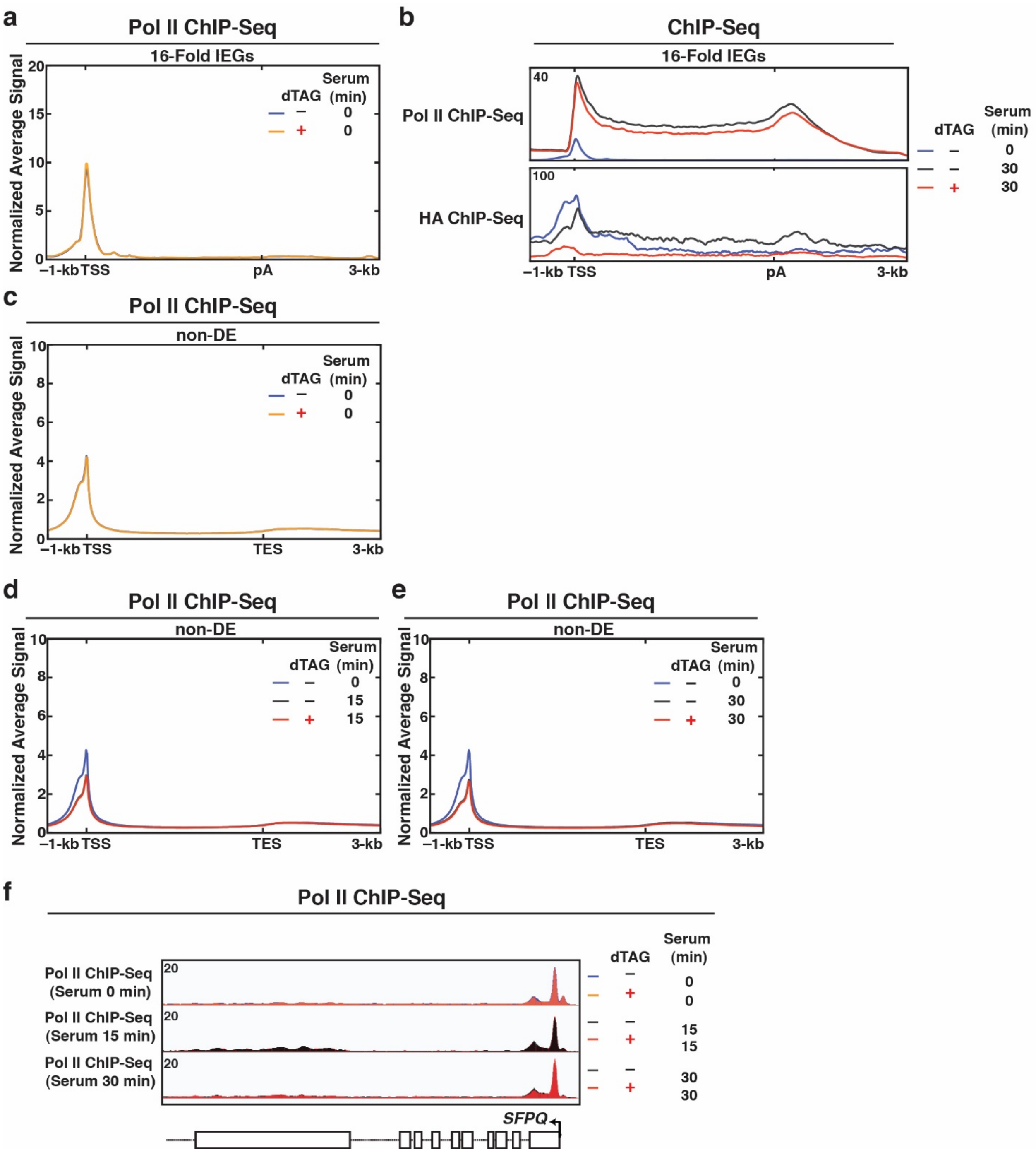

Supplementary Figure 3. KAP1 regulates Pol II occupancy during serum stimulation. a Pol II ChIP-Seq metagene analysis at 16-Fold IEGs in the serum 0 min condition with and without dTAG treatment. See legend for sample identification. **b** Overlayed metagene profiles of Pol II and HA (KAP1) ChIP-Seq in the three indicated conditions. See legend for sample identification. **c-e** Pol II ChIP-Seq metagene profiles at non-DE genes: **c** 0 min, **d** 15 min, **e** and 30 min serum stimulation time points. See legend for sample identification. **f** Pol II ChIP-Seq browser tracks in multiple conditions at one control gene (*SFPQ*). See legend for sample identification.

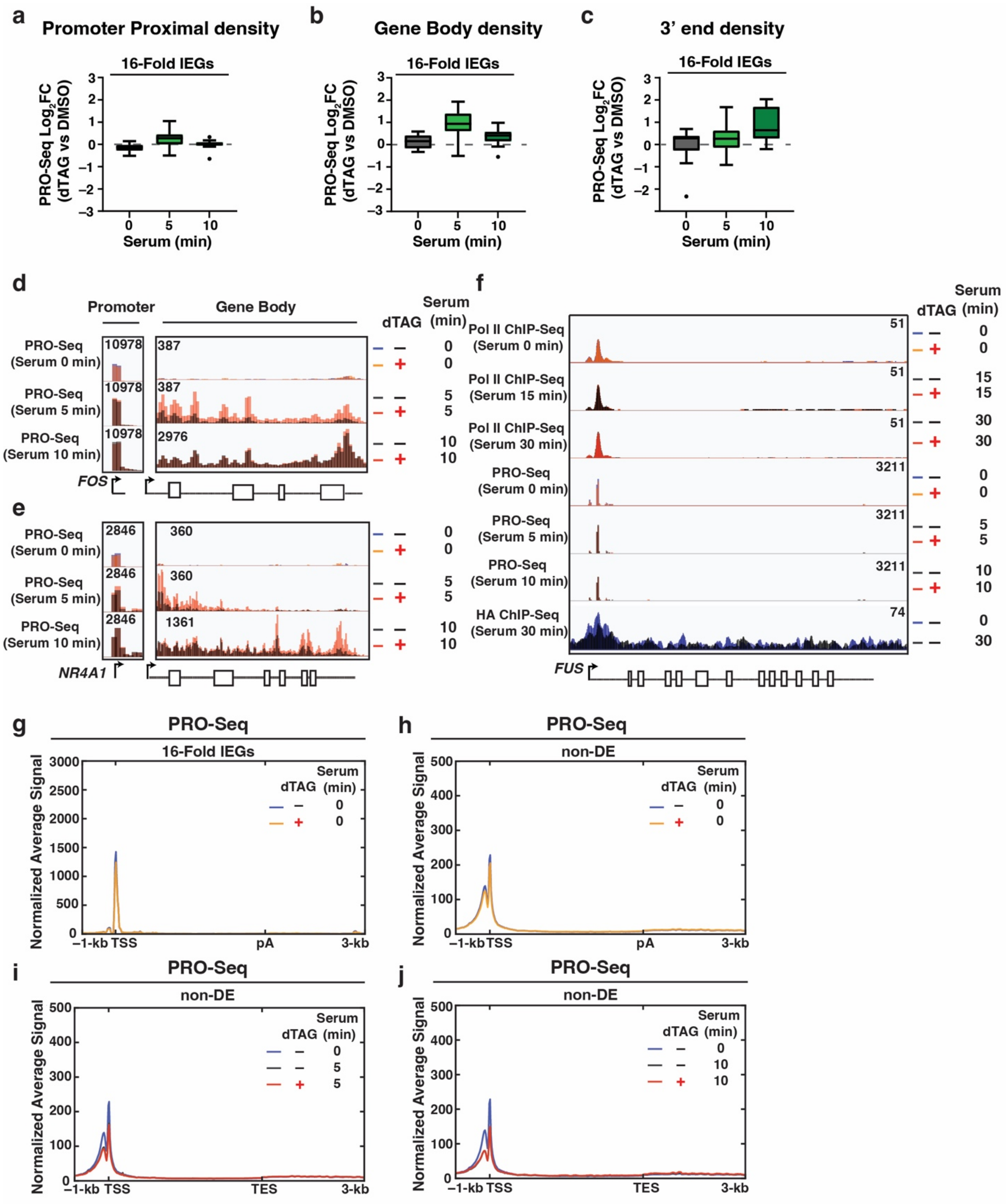

Supplementary Figure 4. Acute KAP1 depletion leads to increased active Pol II density in the gene bodies of IEGs upon serum stimulation. a-c Quantitation's of PRO-Seq signal at 16-Fold IEGs (n=16) at: **a** PP regions, **b** GB regions, and **c** 3' ends. Data represents the Log_2FC value for dTAG versus DMSO at the respective serum time point (see X-axis) using PRO-Seq signal from 2 biological replicates. The n number

represents the number of genes in each cluster plotted. The Tukey plots indicate the median (black center line), the first and third quartiles (edges of the box) and 1.5 × interquartile range below and above the box as whiskers. Dots are presented as genes with normalized signal beyond these defined ranges. **d-e** PRO-Seq browser tracks at multiple time points (see legend for sample identification) at the: **d** *FOS* locus and **e** *NR4A1* locus. **f** Pol II ChIP-Seq, PRO-Seq, and HA ChIP-Seq browser tracks in multiple conditions (see legend for sample identification) at a control gene (*FUS*). **g-h** PRO-Seq metagene analysis at 16-Fold IEGs **g** and non-DE genes **h** in 0 min serum stimulation condition. See legend for sample identification. **i-j** PRO-Seq metagene analysis at non-DE genes at the: **i** 5 min and **j** 10 min serum stimulation time points. See legend for sample identification. Source data are provided as a Source Data file.

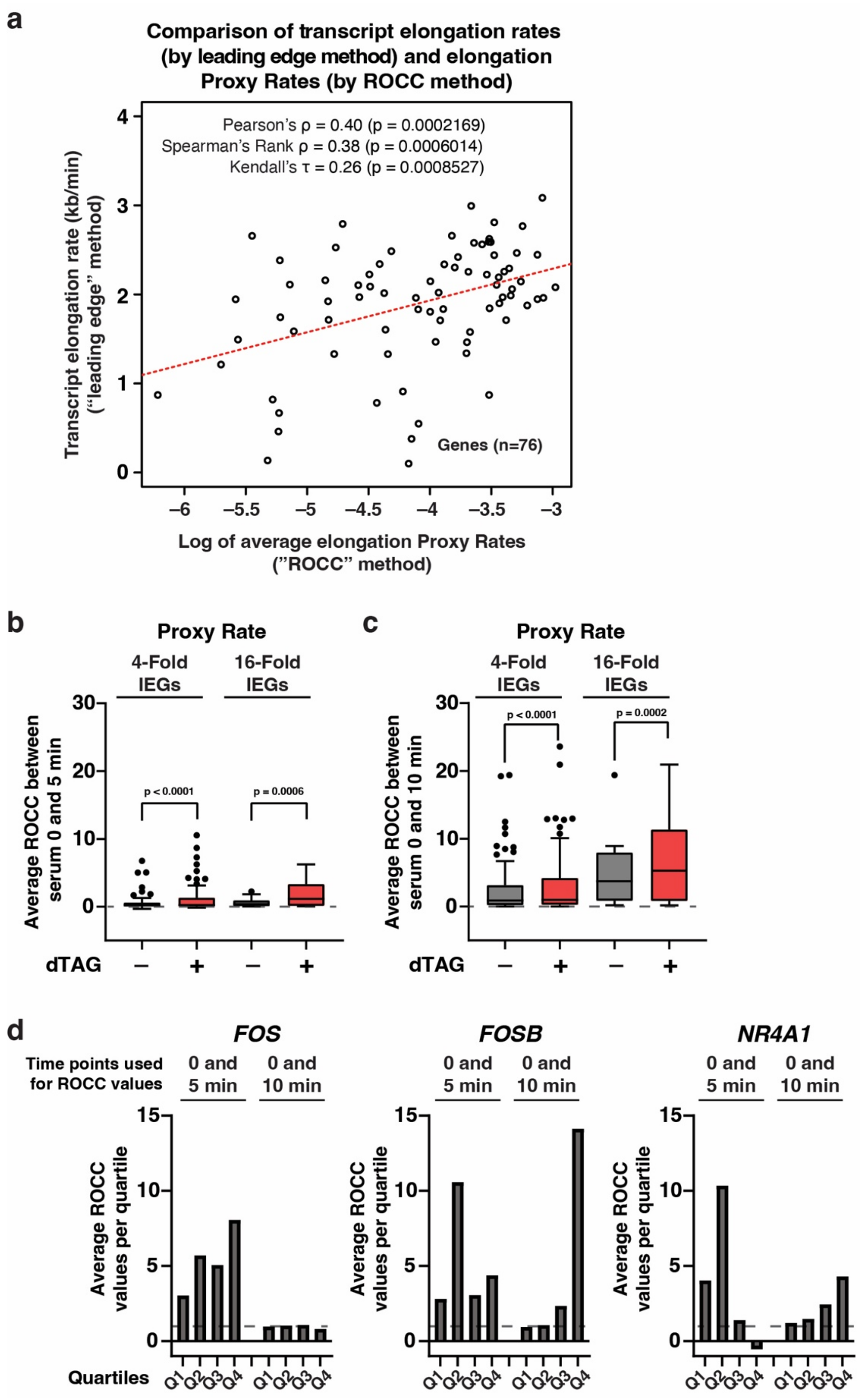

Supplementary Figure 5. Benchmarking of the Proxy Rate analysis using ROCC. a Validation of the ROCC approach to estimate elongation rate using a published estrogen-

inducible gene dataset[1]. The ROCC Proxy Rate analysis was performed as described and tested for correlation to the leading edge method, which captures the wavefront to calculate the elongation rate. Each individual correlation test is labeled, and the P value is indicated. **b-c** Proxy Rate calculations between the **b** serum 0 and 5 min time points, and **c** serum 0 and 10 min time points, separately for 4-Fold IEGs (n=69) and 16-Fold IEGs (n=16). This analysis simply subtracts signal between the designated time point thus allowing the serum 0 to exert maximal influence on the overall rate analysis. Data represents the absolute average ROCC values for the respective sample (see X-axis) using PRO-Seq data from 2 biological replicates. The n number represents the number of genes in each cluster plotted. The Tukey plots indicate the median (black center line), the first and third quartiles (edges of the box) and 1.5 × interquartile range below and above the box as whiskers. Dots are presented as genes with normalized signal beyond these defined ranges. Statistics were calculated between the samples in the plot (dTAG vs. DMSO). Two-sided Wilcoxon signed-rank test. P values are indicated. **d** Proxy Rate calculations for representative IEGs across the four quartiles of gene length. This analysis uses the "single time point" ROCC approach plotted in panels **b** and **c**. The single time points are designated at the top of the graph. Q1: quartile 1, Q2: quartile 2, Q3: quartile 3, Q4: quartile 4. Source data are provided as a Source Data file.

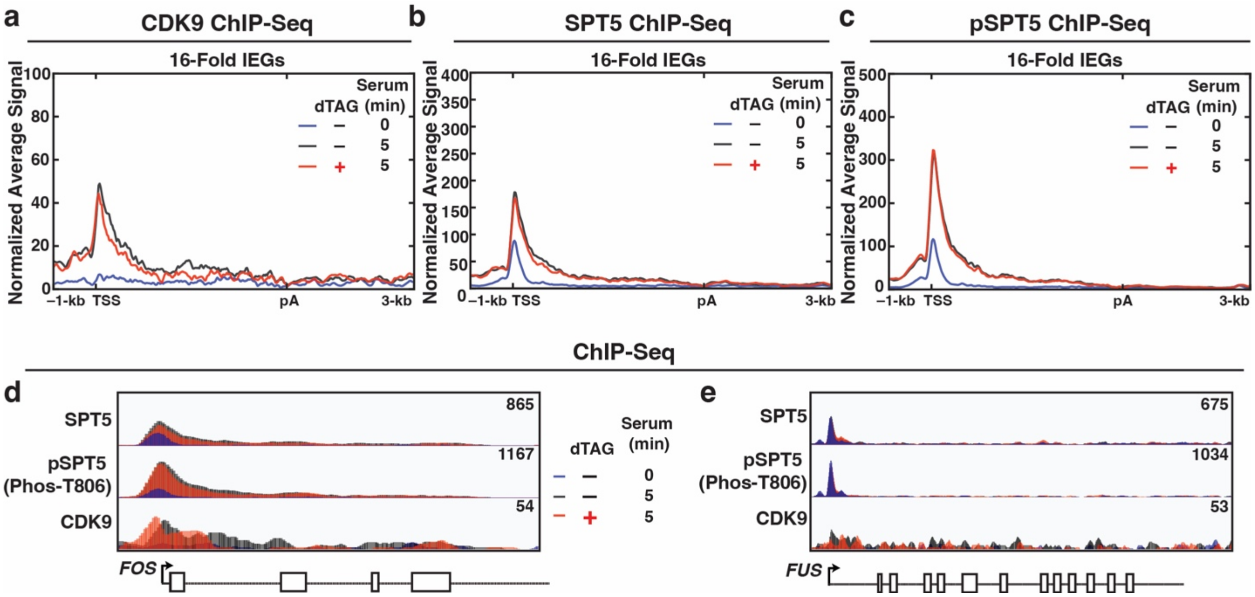

Supplementary Figure 6. Acute KAP1 depletion does not affect the recruitment nor the phosphorylation status of pause release factors. a-c ChIP-Seq metagene profile of: **a** CDK9, **b** total SPT5, and (**c**) pSPT5 (Phos-T806) at 16-Fold IEGs (n=16) in the three indicated conditions. See legend for sample identification. **d-e** CDK9, total SPT5, and pSPT5 browser tracks of: **d** one representative IEG (*FOS*) and **e** one control gene (*FUS*). See legend for sample identification.

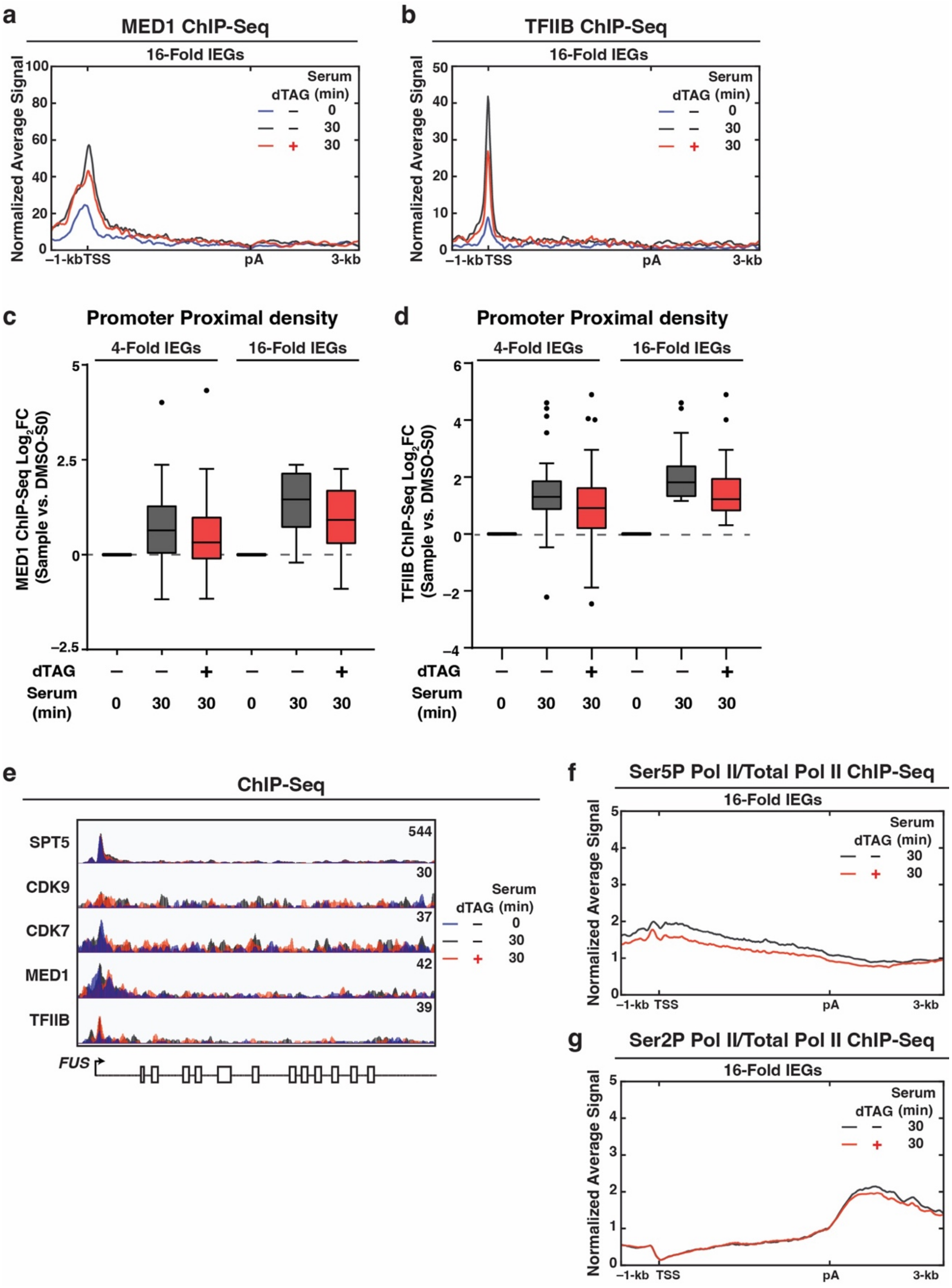

Supplementary Figure 7. Acute KAP1 depletion leads to decreased occupancy of regulators of transcription elongation and initiation. a-b ChIP-Seq metagene analysis

of: **a** MED1 and **b** TFIIB at 16-Fold IEGs (n=16) in the three indicated conditions. See legend for sample identification. **c-d** ChIP-Seq quantitation of: **c** MED1 and **d** TFIIB in PP regions for 4-Fold IEGs (n=69) and 16-Fold IEGs (n=16). Data represents the Log_2FC value for the respective sample (see X-axis) normalized to serum 0 min in DMSO-treated cells using ChIP-Seq signal from one biological replicate. The n number represents the number of genes in each cluster plotted. The Tukey plots indicate the median (black center line), the first and third quartiles (edges of the box) and 1.5 × interquartile range below and above the box as whiskers. Dots are presented as genes with normalized signal beyond these defined ranges. **e** ChIP-Seq browser tracks of all factors in multiple conditions at one control gene (*FUS*). See legend for sample identification. **f-g** ChIP-Seq metagene analysis of: **f** Ser5P Pol II (normalized to total Pol II) and **g** Ser2P Pol II (normalized to total Pol II) at 16-Fold IEGs in DMSO-Serum 30 min and dTAG-Serum 30 min conditions. See legend for sample identification. Source data are provided as a Source Data file.

Supplementary Tables

Supplementary Table 1. Plasmids used in this study.

Plasmid	Origin
pCRIS-PITChv2-C-dTAG-Puro (BRD4)	Addgene 91796
pCRIS-PITChv2-C-TAG-BSD (BRD4)	Addgene 91795
Px330A_sgx _sgPITCh	Universal cutting vector derived from Addgene 58766 and 63670
pCRIS-PITChv2-C-dTAG-Puro (KAP1)	Generated in this study
pCRIS-PITChv2-C-dTAG-BSD (KAP1)	Generated in this study
Px330A_sgx _KAP1C_sgPITCh	Generated in this study

Supplementary Table 2. Primers used in this study.

Number	Name	Sequence (5'-3')	Usage
2691	KAP1 C-Term FWD Guide	CACCGCATGGGGGCTCCAGCCTCAG	dTAG gRNA cloning
2692	KAP1 C-Term REV Guide	AAACCTGAGGCTGGAGCCCCCATGC	dTAG gRNA cloning
2810	KAP1 C-Term gRNA2 PUROBSD FWD	gttccgcgttacatagcatcgtacgcgtacgtgtttggggccctggtgatggcAcctggggtggcggtggctcgggcggtg	dTAG arm cloning
2811	KAP1 C-Term gRNA2 PURO REV	agcattctagagcatcgtacgcgtacgtgtttggGGCCATGGGGGCTCCAGCCTtcaggcaccgggcttgcgggtcatgcaccaggtg	dTAG arm cloning
2812	KAP1 C-Term gRNA2 BSD REV	agcattctagagcatcgtacgcgtacgtgtttggGGCCATGGGGGCTCCAGCCTtcagccctcccacacataaccagagggcagcaat	dTAG arm cloning
2548	pPITChv2_seq fwd	GGGTCATTAGTTCATAGCCC	Sanger sequencing primer for dTAG arm plasmid
2549	pPITChv2_seq rev	TATTAGGAAAGGACAGTGGG	Sanger sequencing primer for dTAG arm plasmid
2550	pX330S_guide_seq	GCTGGCCTTTTGCTCACATG	Sanger sequencing primer for dTAG gRNA plasmid
3286	FOS_RTPCR_FWD	GGGGCAAGGTGGAACAGTTA	RT-qPCR
3287	FOS_RTPCR_REV	AGTTGGTCTGTCTCCGCTTG	RT-qPCR
3656	ATF3_RTPCR_FWD	CGCTGGAATCAGTCACTGTCAG	RT-qPCR
3657	ATF3_RTPCR_REV	CTTGTTTCGGCACTTTGCAGCTG	RT-qPCR
3658	NR4A1_RTPCR_FWD	GGACAACGCTTCATGCCAGCAT	RT-qPCR
3659	NR4A1_RTPCR_REV	CCTTGTTAGCCAGGCAGATGTAC	RT-qPCR
3052	EGR1_ RTPCR_FWD	GGCGAGCAGCCCTACG	RT-qPCR
3053	EGR1_ RTPCR_REV	GCACCTTCTCGTTGTTCAGAG	RT-qPCR
354	RPL19_FWD	ATCGATCGCCACATGTATCA	RT-qPCR
355	RPL19_REV	GCGTGCTTCCTTGGTCTTAG	RT-qPCR
1868	GAPDH_FWD	GCAAATTCCATGGCACCGT	RT-qPCR
1869	GAPDH_REV	TCGCCCCACTTGATTTTGG	RT-qPCR
9	U6_FWD	CTCGCTTCGGCAGCACATATAC	RT-qPCR
10	U6_REV	GGAACGCTTCACGAATTTGCGTG	RT-qPCR
	VRA3 RNA adapter	GAUCGUCGGACUGUAGAACUCUGAAC-/Inverted dT/	PRO-Seq (Purchased from IDT RNase-free HPLC purified)
	VRA5 RNA adapter	CCUUGGCACCCGAGAAUUCCA	See VRA3 usage
	RPI (DNA Oligo)	AATGATACGGCGACCACCGAGATCTACACGTTCAGAGTTCTACAGTCCGA	PRO-Seq (Purchased from IDT PAGE purified)

Supplementary Table 3. Antibodies used in this study.

Target	Vendor	Catalog Number	Assay (Dilution/time)	Figure
KAP1	Abcam	ab22553	Western blot (1:2000/1 hr)	Figure 1 and Supplementary Figure 1
HA	BioLegend	901513	Western blot (1:2000/Overnight)	Figure 1 and Supplementary Figure 1
c-Fos	Cell Signaling Technologies	2250	Western blot (1:1000/Overnight)	Figure 1 and Supplementary Figure 1
ATF3	Santa Cruz Biotechnologies	sc-81189	Western blot (1:1000/Overnight)	Figure 1
Elk-1	Cell Signaling Technologies	9182	Western blot (1:500/Overnight)	Supplementary Figure 1
Elk-1 Phos-S383	Santa Cruz Biotechnologies	sc-8406	Western blot (1:500/Overnight)	Supplementary Figure 1
Actin Rhodamine	Bio-Rad	12004163	Western blot (1:10000/1 hr)	Figure 1 and Supplementary Figure 1
Goat anti-mouse IgG-HRP	Santa Cruz Biotechnologies	sc-2005	Western blot (1:10000/1 hr)	All Figures
Donkey anti-rabbit IgG-HRP	Santa Cruz Biotechnologies	sc-2313	Western blot (1:10000/1 hr)	All Figures
StarBright Blue 700 Goat Anti-Mouse IgG	Bio-Rad	12004158	Western blot (1:10000/1 hr)	All Figures
HA	MilliporeSigma	05-905	ChIP (5 µg/ChIP)	Figure 2
RPB3	MilliporeSigma	ABE999	ChIP (5 µg/ChIP)	Figure 3
SPT5	Bethyl	A300-868A	ChIP (5 µg/ChIP)	Figure 5, Supplementary Figures 6 and 7
CDK9	Cell Signaling Technologies	2316	ChIP (18 µL/ChIP)	Figure 5, Supplementary Figures 6 and 7
SPT5 Phos-T806	Gift from Robert Fisher		ChIP (7 µL/ChIP)	Supplementary Figure 6
TFIIB	Santa Cruz Biotechnologies	sc-271736	ChIP (5 µg/ChIP)	Supplementary Figure 7
MED1	Bethyl	A300-793A	ChIP (5 µg/ChIP)	Supplementary Figure 7
CDK7	Bethyl	A300-405A	ChIP (5 µg/ChIP)	Figure 5
Ser2P Pol II	MilliporeSigma	04-1571	ChIP (10 µg/ChIP)	Supplementary Figure 7
Ser5P Pol II	Active Motif	61085	ChIP (10 µg/ChIP)	Supplementary Figure 7

Supplementary Table 4. Pearson correlation coefficients for all high-throughput sequencing in this study.

Experiment	Number of replicates	Sample**	Pearson's Correlation Coefficient	Figure
TT-Seq	2	DMSO (8hr)	0.99	Supplementary Figure 1
		dTAG (8 hr)	1.00	
HA ChIP-Seq	2	DMSO-S0	0.93	Figure 2 and Supplementary Figure 2
		DMSO-S30	0.83	
		DMSO-S180	0.84	
		dTAG-S30	0.98	
Pol II ChIP-Seq	2	DMSO-S0	0.99	Figure 3 and Supplementary Figure 3
		DMSO-S15	1.00	
		DMSO-S30	0.99	
		dTAG-S0	1.00	
		dTAG-S15	1.00	
		dTAG-S30	0.98	
PRO-Seq	2	DMSO-S0	0.92	Figure 4 and Supplementary Figures 4 and 5
		DMSO-S5	0.96	
		DMSO-S10	0.98	
		dTAG-S0	0.95	
		dTAG-S5	0.96	
		dTAG-S10	0.95	
Ser2P Pol II ChIP-Seq	2	DMSO-S0	1.00	Supplementary Figure 7
		DMSO-S30	1.00	
		dTAG-S30	1.00	

**For TT-Seq, samples were treated with DMSO and dTAG for 8 hr in homeostatic conditions. For all other experiments, cells were treated with serum free media (-S0) or with serum for the indicated time point (for -SX min).

Supplementary Table 5. Alignment statistics for all high-throughput sequencing in this study.

Reads aligning to the human genome (hg38)

Library	Read pairs examined	Read pair duplicates	Percent duplication
PolII_DMSO_Serum0_Replicate1	31139199	6294476	20.3211
PolII_DMSO_Serum0_Replicate2	53092913	11960899	22.6616
PolII_dTAG_Serum0_Replicate1	40657527	8301387	20.5668
PolII_dTAG_Serum0_Replicate2	56973603	11277780	19.9397
PolII_DMSO_Serum15_Replicate1	37015058	7463579	20.2312
PolII_DMSO_Serum15_Replicate2	36368697	8062452	22.2328
PolII_dTAG_Serum15_Replicate1	30761204	6251095	20.3839
PolII_dTAG_Serum15_Replicate2	38423042	8041808	20.9987
PolII_DMSO_Serum30_Replicate1	44639810	11855203	26.607
PolII_DMSO_Serum30_Replicate2	34965948	8353523	23.9364
PolII_dTAG_Serum30_Replicate1	33945856	8432169	24.8867
PolII_dTAG_Serum30_Replicate2	36324858	8703529	24.0072
HA_DMSO_Serum0_Replicate1	40322571	9487295	23.5932
HA_DMSO_Serum0_Replicate2	37981047	9096273	24.0213
HA_DMSO_Serum30_Replicate1	38842070	9006254	23.2523
HA_DMSO_Serum30_Replicate2	36981383	8573790	23.2457
HA_DMSO_Serum180_Replicate1	35130554	7742961	22.0981
HA_DMSO_Serum180_Replicate2	37855181	9125958	24.1659
HA_dTAG_Serum30_Replicate1	33022155	7855107	23.8401
HA_dTAG_Serum30_Replicate2	33463381	8099510	24.2662
PolII_Ser2P_DMSO_Serum0_Replicate1	33022671	8739888	26.5052
PolII_Ser2P_DMSO_Serum0_Replicate2	37910809	10874119	28.7231
PolII_Ser2P_DMSO_Serum30_Replicate1	43140498	14149213	32.8355
PolII_Ser2P_DMSO_Serum30_Replicate2	34302291	10480899	30.5922
PolII_Ser2P_dTAG_Serum30_Replicate1	42211318	15464820	36.6681
PolII_Ser2P_dTAG_Serum30_Replicate2	39556026	13596583	34.4069
PolII_Ser5P_DMSO_Serum0	38517462	9335665	24.3913
PolII_Ser5P_DMSO_Serum30	37912478	7384822	19.5966
PolII_Ser5P_dTAG_Serum30	39801598	7195715	18.1929
SPT5_DMSO_Serum0	24784351	6514407	26.367
SPT5_DMSO_Serum5	25693981	4651674	18.2227
SPT5_dTAG_Serum5	25874588	5152945	20.0323
SPT5_DMSO_Serum30	27387149	5717553	20.9875
SPT5_dTAG_Serum30	26033518	6025396	23.2462
pSPT5_Thr806P_DMSO_Serum0	29791089	11146540	37.4839
pSPT5_Thr806P_DMSO_Serum5	25326385	8855041	35.0331
pSPT5_Thr806P_dTAG_Serum5	25551680	6274354	24.6525
CDK9_DMSO_Serum0	27375650	18760106	68.5339
CDK9_DMSO_Serum5	27834349	14433264	51.886
CDK9_dTAG_Serum5	25574821	16180859	63.2805
CDK9_DMSO_Serum30	22487188	9311695	41.4603
CDK9_dTAG_Serum30	25161269	12385526	49.2601
CDK7_DMSO_Serum0	39339209	14340605	36.5163
CDK7_DMSO_Serum30	34799419	14368796	41.3568
CDK7_dTAG_Serum30	38087252	15085153	39.6802
MED1_DMSO_Serum0	40415656	27890930	69.0142
MED1_DMSO_Serum30	35591514	24323052	68.3405

MED1_dTAG_Serum30	36008872	24138715	67.0311
TFIIB_DMSO_Serum0	20164961	15605622	77.3531
TFIIB_DMSO_Serum30	21264825	12008670	56.4894
TFIIB_dTAG_Serum30	23295485	15770857	67.6928

Spike-in reads aligning to the Drosophila genome (Dm6)

ChIP-Seq library	Read pairs examined	Read pair duplicates	Percent duplication	Spike-in scale factors applied to human files
HA_DMSO_Serum0_R1	36323	7994	39.084	1.251
HA_DMSO_Serum0_R2	40994	9010	38.5216	1.251
HA_DMSO_Serum30_R1	36214	7482	38.6249	1.251
HA_DMSO_Serum30_R2	21170	4249	42.2422	1.3335
HA_DMSO_Serum180_R1	15691	3128	43.0812	1.3643
HA_DMSO_Serum180_R2	18918	4022	43.4092	1.3335
HA_dTAG_Serum30_R1	147108	32323	26.2654	0.4548
HA_dTAG_Serum30_R2	167175	37739	26.6953	0.6821
SPT5_DMSO_Serum0	79289	21707	32.2842	0.7579
SPT5_DMSO_Serum5	29941	5183	32.0865	1.1487
SPT5_dTAG_Serum5	27491	5339	34.4706	1.1789
SPT5_DMSO_Serum30	40127	8249	33.569	1.1487
SPT5_dTAG_Serum30	43116	9935	34.0166	1
pSPT5_Thr806P_DMSO_Serum0	308173	126015	41.9428	0.7937
pSPT5_Thr806P_DMSO_Serum5	99482	36701	40.1875	1.2114
pSPT5_Thr806P_dTAG_Serum5	95869	24542	30.8381	1.1447
CDK9_DMSO_Serum0	452080	333921	73.5347	1.0073
CDK9_DMSO_Serum5	245142	135489	55.8562	1
CDK9_dTAG_Serum5	233371	155879	66.6648	1.2941
CDK9_DMSO_Serum30	210504	96449	46.8914	0.9441
CDK9_dTAG_Serum30	306263	165895	54.6788	0.7841
CDK7_DMSO_Serum0	112910	44695	44.4832	1
CDK7_DMSO_Serum30	130406	56403	47.1684	1
CDK7_dTAG_Serum30	156589	65781	46.2314	1
MED1_DMSO_Serum0	561891	404285	72.9469	0.9103
MED1_DMSO_Serum30	505410	358961	71.6832	0.951
MED1_dTAG_Serum30	450515	316463	70.6458	1
TFIIB_DMSO_Serum0	189012	148324	77.4249	1
TFIIB_DMSO_Serum30	186992	116588	62.159	0.9086
TFIIB_dTAG_Serum30	115949	82997	70.4418	1.1447

Supplementary References

1 Danko, C. G. *et al.* Signaling pathways differentially affect RNA polymerase II initiation, pausing, and elongation rate in cells. *Mol Cell* **50**, 212-222 (2013). https://doi.org/10.1016/j.molcel.2013.02.015
