# Supplementary material for: KAP1 negatively regulates RNA polymerase II elongation kinetics to activate signal-induced transcription
Source: Nat Commun. 2024 Jul 12;15:5859. doi: 10.1038/s41467-024-49905-7 (PMC11245487; doi:10.1038/s41467-024-49905-7)
Supplement: Supplementary file 3 — Description of Additional Supplementary Files [file 41467_2024_49905_MOESM3_ESM.pdf]

File name: Supplementary Data 1

Description: List of differentially expressed genes using RNA Seq. and TT-Seq.
