# Supplementary material for: KAP1 negatively regulates RNA polymerase II elongation kinetics to activate signal-induced transcription
Source: Nat Commun. 2024 Jul 12;15:5859. doi: 10.1038/s41467-024-49905-7 (PMC11245487; doi:10.1038/s41467-024-49905-7)
Supplement: Supplementary file 5 — Reporting summary [file 41467_2024_49905_MOESM5_ESM.pdf]

nature portfolio

Corresponding author(s): Ivan D'Orso

Last updated by author(s): Jun 7, 2024

Reporting Summary

Nature Portfolio wishes to improve the reproducibility of the work that we publish. This form provides structure for consistency and transparency in reporting. For further information on Nature Portfolio policies, see our Editorial Policies and the Editorial Policy Checklist.

Statistics

For all statistical analyses, confirm that the following items are present in the figure legend, table legend, main text, or Methods section.

n/a	Confirmed	
☐	☒	The exact sample size (*n*) for each experimental group/condition, given as a discrete number and unit of measurement
☐	☒	A statement on whether measurements were taken from distinct samples or whether the same sample was measured repeatedly
☐	☒	The statistical test(s) used AND whether they are one- or two-sided<br>*Only common tests should be described solely by name; describe more complex techniques in the Methods section.*
☒	☐	A description of all covariates tested
☒	☐	A description of any assumptions or corrections, such as tests of normality and adjustment for multiple comparisons
☐	☒	A full description of the statistical parameters including central tendency (e.g. means) or other basic estimates (e.g. regression coefficient) AND variation (e.g. standard deviation) or associated estimates of uncertainty (e.g. confidence intervals)
☐	☒	For null hypothesis testing, the test statistic (e.g. *F*, *t*, *r*) with confidence intervals, effect sizes, degrees of freedom and *P* value noted<br>*Give P values as exact values whenever suitable.*
☒	☐	For Bayesian analysis, information on the choice of priors and Markov chain Monte Carlo settings
☒	☐	For hierarchical and complex designs, identification of the appropriate level for tests and full reporting of outcomes
☐	☒	Estimates of effect sizes (e.g. Cohen's *d*, Pearson's *r*), indicating how they were calculated

Our web collection on statistics for biologists contains articles on many of the points above.

Software and code

Policy information about availability of computer code

Data collection	No software was used to 'collect' data. All data was generated in this manuscript and analyzed using the indicated softwares/programs. Versions are included below to describe the versions used for each software.
Data analysis	Detailed scripts and commands used for all NGS analysis in this paper have been deposited in the D'Orso Lab public GitLab (https://git.biohpc.swmed.edu/ivandorsolab/hyder-et-al._-2024). Briefly, data analysis tools utilized for each genomics experiment are described below.<br><br>RNA-Seq/TT-Seq: Briefly, raw fastq data files were ran through fastqc/0.11.8 and low quality reads/adapter contaminations were removed using trimgalore/0.6.4. Reads were mapped to the hg38 human reference genome using star/2.7.3a. Spike-ins were mapped to a fasta file of ERCC sequences provided by the manufacturer. featureCounts (subread/1.6.3) was used to calculate counts across the entire gene of protein coding genes using a .gtf file containing all protein coding genes as well as ERCC spike-ins. EDASeq/2.32.0, RUVSeq/1.32.0, and EdgeR/3.40.1 commands were used for spike-in normalization and differential gene expression analysis of genes that contained at least 10 counts in 6 samples (RNA-Seq) and 50 counts in 4 samples (TT-Seq) was performed. Volcano plots were made using EnhancedVolcano/1.16.0 from bioconductor.<br><br>ChIP-Seq: Briefly, raw fastq data files were ran through fastqc/0.11.8 and low quality reads/adaptor contaminations were removed using trimgalore/0.6.4. Reads were mapped to the hg38 human reference genome using bowtie2/2.4.2. Reads were also mapped to the dm6 Drosophila genome to extract reads originating from the spike-in chromatin for ChIPs that were normalized using spike-ins. Duplicates were marked and removed using picard/2.10.3 and then files were sorted and indexed using samtools/1.6 in preparation for bigWig generation. Normalized bigWig files were made from sorted bam files using the bamCoverage command using deepTools/2.3.5.

PRO-Seq: Briefly, raw fastq data files were ran through fastqc/0.11.8 and low quality reads/adaptor contaminations were removed using Cutadapt/2.5. Reads were subsequently aligned to the human reference genome hg38 using BWA/0.7.5. The aligned bam files were converted into RPKM-normalized bigWig format using deepTools/2.3.5 and bedGraphToBigWig program to visualize in IGV.

For manuscripts utilizing custom algorithms or software that are central to the research but not yet described in published literature, software must be made available to editors and reviewers. We strongly encourage code deposition in a community repository (e.g. GitHub). See the Nature Portfolio guidelines for submitting code & software for further information.

Data

Policy information about availability of data

All manuscripts must include a data availability statement. This statement should provide the following information, where applicable:

- Accession codes, unique identifiers, or web links for publicly available datasets
- A description of any restrictions on data availability
- For clinical datasets or third party data, please ensure that the statement adheres to our policy

Data Availability. Original western blot data generated in this study have been deposited in the Mendeley database (https://data.mendeley.com/datasets/ffsbjbkkvx/1). Raw NGS data generated in this study have been deposited at NCBI GEO under accession number GSE246218 (https://www.ncbi.nlm.nih.gov/geo/query/acc.cgi?acc=GSE246218). Source data are provided with this paper.

Code Availability. Detailed scripts and commands used for all NGS analysis in this paper have been deposited in the D'Orso Lab public GitLab (https://git.biohpc.swmed.edu/ivandorsolab/hyder-et-al._-2024).

Research involving human participants, their data, or biological material

Policy information about studies with human participants or human data. See also policy information about sex, gender (identity/presentation), and sexual orientation and race, ethnicity and racism.

Reporting on sex and gender

Use the terms sex (biological attribute) and gender (shaped by social and cultural circumstances) carefully in order to avoid confusing both terms. Indicate if findings apply to only one sex or gender; describe whether sex and gender were considered in study design; whether sex and/or gender was determined based on self-reporting or assigned and methods used.
Provide in the source data disaggregated sex and gender data, where this information has been collected, and if consent has been obtained for sharing of individual-level data; provide overall numbers in this Reporting Summary. Please state if this information has not been collected.
Report sex- and gender-based analyses where performed, justify reasons for lack of sex- and gender-based analysis.

Reporting on race, ethnicity, or other socially relevant groupings

Please specify the socially constructed or socially relevant categorization variable(s) used in your manuscript and explain why they were used. Please note that such variables should not be used as proxies for other socially constructed/relevant variables (for example, race or ethnicity should not be used as a proxy for socioeconomic status).
Provide clear definitions of the relevant terms used, how they were provided (by the participants/respondents, the researchers, or third parties), and the method(s) used to classify people into the different categories (e.g. self-report, census or administrative data, social media data, etc.)
Please provide details about how you controlled for confounding variables in your analyses.

Population characteristics

Describe the covariate-relevant population characteristics of the human research participants (e.g. age, genotypic information, past and current diagnosis and treatment categories). If you filled out the behavioural & social sciences study design questions and have nothing to add here, write "See above."

Recruitment

Describe how participants were recruited. Outline any potential self-selection bias or other biases that may be present and how these are likely to impact results.

Ethics oversight

Identify the organization(s) that approved the study protocol.

Note that full information on the approval of the study protocol must also be provided in the manuscript.

Field-specific reporting

Please select the one below that is the best fit for your research. If you are not sure, read the appropriate sections before making your selection.

☒ Life sciences ☐ Behavioural & social sciences ☐ Ecological, evolutionary & environmental sciences

For a reference copy of the document with all sections, see nature.com/documents/nr-reporting-summary-flat.pdf

Life sciences study design

All studies must disclose on these points even when the disclosure is negative.

Sample size

No statistical method was done to determine sample size. For RT-qPCR assays and RNA-Seq, three replicates were used. For genomics experiments (TT-Seq, ChIP-Seq, PRO-Seq) two replicates were performed and sequenced. For some ChIP-Seqs, only one replicate was performed (described in the methods), but data crossvalidated by -qPCR.

Data exclusions

To address reviewer requests asking for spike-in normalization for ChIP-Seq data, ChIP-Seqs for SPT5, CDK9, CDK7, TFIIB, and MED1 in Figure 5 of this manuscript were repeated to include spike-ins in the data analysis (originally these were normalized to read depth only). Importantly, replication of the experiment and analysis using both read depth and spike-in normalization yielded very similar results suggesting that occupancy of these factors were decreased at late serum stimulation time points (30 min) upon acute KAP1 depletion. Thus, as requested by one of the reviewer's, only the spike-in normalized datasets were included in the manuscript and the original ChIP-Seq's were excluded. Other than this, no data was excluded from the manuscript.

Replication

Western blots were performed at least 2 times to ensure phenotypes were robust. RNA-Seq, TT-Seq, and ChIP-Seq have all been validated by -qPCR based approaches (RT-qPCR, ChIP-qPCR, etc.) on different days to ensure reproducibility of all genomics datasets.

Randomization

No randomization was conducted.

Blinding

No blinding was conducted.

Reporting for specific materials, systems and methods

We require information from authors about some types of materials, experimental systems and methods used in many studies. Here, indicate whether each material, system or method listed is relevant to your study. If you are not sure if a list item applies to your research, read the appropriate section before selecting a response.

Materials & experimental systems

n/a	Involved in the study
☐	☒ Antibodies
☐	☒ Eukaryotic cell lines
☒	☐ Palaeontology and archaeology
☒	☐ Animals and other organisms
☒	☐ Clinical data
☒	☐ Dual use research of concern
☒	☐ Plants

Methods

n/a	Involved in the study
☐	☒ ChIP-seq
☒	☐ Flow cytometry
☒	☐ MRI-based neuroimaging

Antibodies

Antibodies used

Target/Vendor/Catalog Number/Assay (Dilution/time)
KAP1/Abcam/ab22553/Western blot (1:2000/1 hr)
HA/BioLegend/901513/Western blot (1:2000/Overnight)
c-Fos/Cell Signaling Technologies/2250/Western blot (1:1000/Overnight)
ATF3/Santa Cruz Biotechnologies/sc-81189/Western blot (1:1000/Overnight)
Elk-1/Cell Signaling Technologies/9182/Western blot (1:500/Overnight)
Elk-1 Phos-S383/Santa Cruz Biotechnologies/sc-8406/Western blot (1:500/Overnight)
Actin Rhodamine/Bio-Rad/12004163/Western blot (1:10000/1 hr)
Goat anti-mouse IgG-HRP/Santa Cruz Biotechnologies/sc-2005/Western blot (1:10000/1 hr)
Donkey anti-rabbit IgG-HRP/Santa Cruz Biotechnologies/sc-2313/Western blot (1:10000/1 hr)
StarBright Blue 700 Goat Anti-Mouse IgG/Bio-Rad/12004158/Western blot (1:10000/1 hr)
HA/Millipore/05-905/ChIP (5 μg/ChIP)
RPB3/Millipore/ABE999/ChIP (5 μg/ChIP)
SPT5/Bethyl/Thermo/A300-868A/ChIP (5 μg/ChIP)
pSPT5/gift_Robert_Fisher/ChIP/ (7 uL/ChIP)
CDK9/Cell Signaling Technologies/2316/ChIP (18 μL/ChIP)
pS2 Pol II/Millipore/04-1571/ChIP (10 μg/ChIP)
pS5 Pol II/Active Motif/61085/ChIP (10 μg/ChIP)
TFIIB/Santa Cruz Biotechnologies/sc-271736/ChIP (5 μg/ChIP)
MED1/Bethyl/A300-793A/ChIP (5 μg/ChIP)
CDK7/Bethyl/A300-405A/ChIP (5 μg/ChIP)

Validation

All antibodies were validated by western blot to ensure specificity of the antibody binding the expected antigen and presence of a band at the correct molecular weight according to the vendors recommendations. For KAP1, antibodies were validated additionally by probing in both KAP1-expressing and -depleted conditions. Antibodies for ChIP-Seq were additionally validated by ChIP-Seq by ensuring that chromatin profiling at the expected sites was observed. In the case of this paper, we monitored factor occupancy at Immediate Early Gene (IEGs), where most factors in this study were recruited to IEGs upon serum stimulation. KAP1 occupancy was validated by ChIP-Seq additionally when cells do not express KAP1 for specific targeting.

Eukaryotic cell lines

Policy information about cell lines and Sex and Gender in Research

Cell line source(s)

HCT116 cells (CCL-247) were originally purchased from ATCC (https://www.atcc.org/products/ccl-247)

Authentication

Cell lines were not authenticated.

Mycoplasma contamination

All cell lines were confirmed negative for mycoplasma testing using a kit from Southern biotech (catalog 13100-01). Cell lines were validated mycoplasma-negative every 3-6 months.

Commonly misidentified lines
(See ICLAC register)

NA

Plants

Seed stocks

Report on the source of all seed stocks or other plant material used. If applicable, state the seed stock centre and catalogue number. If plant specimens were collected from the field, describe the collection location, date and sampling procedures.

Novel plant genotypes

Describe the methods by which all novel plant genotypes were produced. This includes those generated by transgenic approaches, gene editing, chemical/radiation-based mutagenesis and hybridization. For transgenic lines, describe the transformation method, the number of independent lines analyzed and the generation upon which experiments were performed. For gene-edited lines, describe the editor used, the endogenous sequence targeted for editing, the targeting guide RNA sequence (if applicable) and how the editor was applied.

Authentication

Describe any authentication procedures for each seed stock used or novel genotype generated. Describe any experiments used to assess the effect of a mutation and, where applicable, how potential secondary effects (e.g. second site T-DNA insertions, mosiacism, off-target gene editing) were examined.

ChIP-seq

Data deposition

☒ Confirm that both raw and final processed data have been deposited in a public database such as GEO.

☐ Confirm that you have deposited or provided access to graph files (e.g. BED files) for the called peaks.

Data access links
May remain private before publication.

Raw NGS datasets have been deposited at NCBI GEO under accession number GSE246218.

Files in database submission

file name 1 file name 2
RPB3_ChIP-DMSO-SS_R1_S33_R1_001.fastq.gz RPB3_ChIP-DMSO-SS_R1_S33_R2_001.fastq.gz
RPB3_ChIP-DMSO-SS_R2_S34_R1_001.fastq.gz RPB3_ChIP-DMSO-SS_R2_S34_R2_001.fastq.gz
RPB3_ChIP-dTAG-SS_R1_S35_R1_001.fastq.gz RPB3_ChIP-dTAG-SS_R1_S35_R2_001.fastq.gz
RPB3_ChIP-dTAG-SS_R2_S36_R1_001.fastq.gz RPB3_ChIP-dTAG-SS_R2_S36_R2_001.fastq.gz
DS15_RPB3_R1_S5_R1_001.fastq.gz DS15_RPB3_R1_S5_R2_001.fastq.gz
DS15_RPB3_R2_S6_R1_001.fastq.gz DS15_RPB3_R2_S6_R2_001.fastq.gz
dTAGS15_RPB3_R1_S7_R1_001.fastq.gz dTAGS15_RPB3_R1_S7_R2_001.fastq.gz
dTAGS15_RPB3_R2_S8_R1_001.fastq.gz dTAGS15_RPB3_R2_S8_R2_001.fastq.gz
DMSO-S30_R1_RPB3_S13_R1_001.fastq DMSO-S30_R1_RPB3_S13_R2_001.fastq
DMSO-S30_R2_RPB3_S14_R1_001.fastq DMSO-S30_R2_RPB3_S14_R2_001.fastq
dTAG-S30_R1_RPB3_S16_R1_001.fastq dTAG-S30_R1_RPB3_S16_R2_001.fastq
dTAG-S30_R2_RPB3_S15_R1_001.fastq dTAG-S30_R2_RPB3_S15_R2_001.fastq
D-S30-HA-R1_S25_R1_001.fastq.gz D-S30-HA-R1_S25_R2_001.fastq.gz
D-S30-HA-R2_S26_R1_001.fastq.gz D-S30-HA-R2_S26_R2_001.fastq.gz
D-S3H-HA-R1_S27_R1_001.fastq.gz D-S3H-HA-R1_S27_R2_001.fastq.gz
D-S3H-HA-R2_S28_R1_001.fastq.gz D-S3H-HA-R2_S28_R2_001.fastq.gz
D-SS-HA-R1_S23_R1_001.fastq.gz D-SS-HA-R1_S23_R2_001.fastq.gz
D-SS-HA-R2_S24_R1_001.fastq.gz D-SS-HA-R2_S24_R2_001.fastq.gz
dTAG-S30-HA-R1_S29_R1_001.fastq.gz dTAG-S30-HA-R1_S29_R2_001.fastq.gz
dTAG-S30-HA-R2_S30_R1_001.fastq.gz dTAG-S30-HA-R2_S30_R2_001.fastq.gz
DS30_S2P_R1_S11_R1_001.fastq.gz DS30_S2P_R1_S11_R2_001.fastq.gz
DS30_S2P_R2_S12_R1_001.fastq.gz DS30_S2P_R2_S12_R2_001.fastq.gz
DSS_S2P_R1_S9_R1_001.fastq.gz DSS_S2P_R1_S9_R2_001.fastq.gz
DSS_S2P_R2_S10_R1_001.fastq.gz DSS_S2P_R2_S10_R2_001.fastq.gz
dTAG_S30_S2P_R1_S15_R1_001.fastq.gz dTAG_S30_S2P_R1_S15_R2_001.fastq.gz
dTAG_S30_S2P_R2_S16_R1_001.fastq.gz dTAG_S30_S2P_R2_S16_R2_001.fastq.gz
DS30_Ser5P_Pol_II_S33_R1_001.fastq.gz DS30_Ser5P_Pol_II_S33_R2_001.fastq.gz
DSS_Ser5P_Pol_II_S32_R1_001.fastq.gz DSS_Ser5P_Pol_II_S32_R2_001.fastq.gz
dTAG_S30_Ser5P_Pol_II_S34_R1_001.fastq.gz dTAG_S30_Ser5P_Pol_II_S34_R2_001.fastq.gz
DS0_CDK7_R1_S1_R1_001.fastq.gz DS0_CDK7_R1_S1_R2_001.fastq.gz
DS0_MED1_R1_S2_R1_001.fastq.gz DS0_MED1_R1_S2_R2_001.fastq.gz
DS30_CDK7_R1_S9_R1_001.fastq.gz DS30_CDK7_R1_S9_R2_001.fastq.gz
DS30_CDK9_R1_S17_R1_001.fastq.gz DS30_CDK9_R1_S17_R2_001.fastq.gz
DS30_MED1_R1_S10_R1_001.fastq.gz DS30_MED1_R1_S10_R2_001.fastq.gz
DS30_SPT5_R1_S8_R1_001.fastq.gz DS30_SPT5_R1_S8_R2_001.fastq.gz
DS30_TFIIB_R1_S33_R1_001.fastq.gz DS30_TFIIB_R1_S33_R2_001.fastq.gz
DS5_CDK9_R1_S13_R1_001.fastq.gz DS5_CDK9_R1_S13_R2_001.fastq.gz
DS5_pSPT5_R1_S24_R1_001.fastq.gz DS5_pSPT5_R1_S24_R2_001.fastq.gz

DS5_SPT5_R1_S4_R1_001.fastq.gz DS5_SPT5_R1_S4_R2_001.fastq.gz
DSS_CDK9_R1_S11_R1_001.fastq.gz DSS_CDK9_R1_S11_R2_001.fastq.gz
DSS_pSPT5_R1_S22_R1_001.fastq.gz DSS_pSPT5_R1_S22_R2_001.fastq.gz
DSS_SPT5_R1_S2_R1_001.fastq.gz DSS_SPT5_R1_S2_R2_001.fastq.gz
DSS_TFIIB_R1_S32_R1_001.fastq.gz DSS_TFIIB_R1_S32_R2_001.fastq.gz
dTS30_CDK7_R1_S12_R1_001.fastq.gz dTS30_CDK7_R1_S12_R2_001.fastq.gz
dTS30_CDK9_R1_S19_R1_001.fastq.gz dTS30_CDK9_R1_S19_R2_001.fastq.gz
dTS30_MED1_R1_S13_R1_001.fastq.gz dTS30_MED1_R1_S13_R2_001.fastq.gz
dTS30_SPT5_R1_S10_R1_001.fastq.gz dTS30_SPT5_R1_S10_R2_001.fastq.gz
dTS30_TFIIB_R1_S34_R1_001.fastq.gz dTS30_TFIIB_R1_S34_R2_001.fastq.gz
dTS5_CDK9_R1_S15_R1_001.fastq.gz dTS5_CDK9_R1_S15_R2_001.fastq.gz
dTS5_pSPT5_R1_S26_R1_001.fastq.gz dTS5_pSPT5_R1_S26_R2_001.fastq.gz
dTS5_SPT5_R1_S6_R1_001.fastq.gz dTS5_SPT5_R1_S6_R2_001.fastq.gz

Genome browser session
(e.g. UCSC)

No longer applicable

Methodology

Replicates

For HA, Pol II, and Ser2P Pol II ChIP-Seq data, two biological replicates were analyzed independently until the bigWig stage, and were merged after visual inspection of individual browser tracks in IGV and Pearson correlation analysis using deepTools (see Supplementary Table 4 for Pearson correlation coefficients). One replicate of each antibody in each condition was performed for the following ChIP-Seqs: SPT5, pSPT5, CDK9, CDK7, TFIIB, MED1, and Ser5P Pol II. All data was validated in at least 2 regions of the genome by ChIP-qPCR.

Sequencing depth

All ChIP-Seq experiments utilized 50-base pair long, paired end reads. All ChIP-Seqs were sequenced to obtain roughly ~33,000,000 reads per sample. Below is a table of Picard metrics (Duplication mapping) listing each ChIP-Seq sample, the total number of read pairs examined, number of read pair duplicates, read pair optical duplicates, and percent duplication.

Human Alignment Statistics
Library READ_PAIRS_EXAMINED READ_PAIR_DUPLICATES READ_PAIR_OPTICAL_DUPLICATES PERCENT_DUPLICATION
PolII_DMSO_Serum0_Replicate1 31139199 6294476 301836 20.3211
PolII_DMSO_Serum0_Replicate2 53092913 11960899 616571 22.6616
PolII_dTAG_Serum0_Replicate1 40657527 8301387 395492 20.5668
PolII_dTAG_Serum0_Replicate2 56973603 11277780 574120 19.9397
PolII_DMSO_Serum15_Replicate1 37015058 7463579 368326 20.2312
PolII_DMSO_Serum15_Replicate2 36368697 8062452 403589 22.2328
PolII_dTAG_Serum15_Replicate1 30761204 6251095 314708 20.3839
PolII_dTAG_Serum15_Replicate2 38423042 8041808 392821 20.9987
PolII_DMSO_Serum30_Replicate1 44639810 11855203 611282 26.607
PolII_DMSO_Serum30_Replicate2 34965948 8353523 430721 23.9364
PolII_dTAG_Serum30_Replicate1 33945856 8432169 447114 24.8867
PolII_dTAG_Serum30_Replicate2 36324858 8703529 456197 24.0072
HA_DMSO_Serum0_Replicate1 40322571 9487295 517979 23.5932
HA_DMSO_Serum0_Replicate2 37981047 9096273 479038 24.0213
HA_DMSO_Serum30_Replicate1 38842070 9006254 497310 23.2523
HA_DMSO_Serum30_Replicate2 36981383 8573790 467850 23.2457
HA_DMSO_Serum180_Replicate1 35130554 7742961 444722 22.0981
HA_DMSO_Serum180_Replicate2 37855181 9125958 546770 24.1659
HA_dTAG_Serum30_Replicate1 33022155 7855107 382585 23.8401
HA_dTAG_Serum30_Replicate2 33463381 8099510 377468 24.2662
PolII_Ser2P_DMSO_Serum0_Replicate1 33022671 8739888 339534 26.5052
PolII_Ser2P_DMSO_Serum0_Replicate2 37910809 10874119 441413 28.7231
PolII_Ser2P_DMSO_Serum30_Replicate1 43140498 14149213 472258 32.8355
PolII_Ser2P_DMSO_Serum30_Replicate2 34302291 10480899 373169 30.5922
PolII_Ser2P_dTAG_Serum30_Replicate1 42211318 15464820 446957 36.6681
PolII_Ser2P_dTAG_Serum30_Replicate2 39556026 13596583 376509 34.4069
PolII_Ser5P_DMSO_Serum0 38517462 9335665 348369 24.3913
PolII_Ser5P_DMSO_Serum30 37912478 7384822 303027 19.5966
PolII_Ser5P_dTAG_Serum30 39801598 7195715 317036 18.1929
SPT5_DMSO_Serum0 24784351 6514407 178569 26.367
SPT5_DMSO_Serum5 25693981 4651674 178579 18.2227
SPT5_dTAG_Serum5 25874588 5152945 190592 20.0323
SPT5_DMSO_Serum30 27387149 5717553 185709 20.9875
SPT5_dTAG_Serum30 26033518 6025396 196893 23.2462
pSPT5_Thr806P_DMSO_Serum0 29791089 11146540 201191 37.4839
pSPT5_Thr806P_DMSO_Serum5 25326385 8855041 161381 35.0331
pSPT5_Thr806P_dTAG_Serum5 25551680 6274354 154688 24.6525
CDK9_DMSO_Serum0 27375650 18760106 193159 68.5339
CDK9_DMSO_Serum5 27834349 14433264 185643 51.886
CDK9_dTAG_Serum5 25574821 16180859 171514 63.2805
CDK9_DMSO_Serum30 22487188 9311695 135878 41.4603
CDK9_dTAG_Serum30 25161269 12385526 145542 49.2601
CDK7_DMSO_Serum0 39339209 14340605 364074 36.5163
CDK7_DMSO_Serum30 34799419 14368796 314666 41.3568

CDK7_dTAG_Serum30 38087252 15085153 363032 39.6802
MED1_DMSO_Serum0 40415656 27890930 390585 69.0142
MED1_DMSO_Serum30 35591514 24323052 365057 68.3405
MED1_dTAG_Serum30 36008872 24138715 338116 67.0311
TFIIB_DMSO_Serum0 20164961 15605622 136576 77.3531
TFIIB_DMSO_Serum30 21264825 12008670 127737 56.4894
TFIIB_dTAG_Serum30 23295485 15770857 149489 67.6928

Spike-in Alignment Statistics
Library READ_PAIRS_EXAMINED READ_PAIR_DUPLICATES READ_PAIR_OPTICAL_DUPLICATES PERCENT_DUPLICATION Spike-in Scale Factors applied to human files
HA_DMSO_Serum0_Replicate1 36323 7994 380 39.084 1.251
HA_DMSO_Serum0_Replicate2 40994 9010 487 38.5216 1.251
HA_DMSO_Serum30_Replicate1 36214 7482 400 38.6249 1.251
HA_DMSO_Serum30_Replicate2 21170 4249 231 42.2422 1.3335
HA_DMSO_Serum180_Replicate1 15691 3128 158 43.0812 1.3643
HA_DMSO_Serum180_Replicate2 18918 4022 221 43.4092 1.3335
HA_dTAG_Serum30_Replicate1 147108 32323 1483 26.2654 0.4548
HA_dTAG_Serum30_Replicate2 167175 37739 1615 26.6953 0.6821
SPT5_DMSO_Serum0 79289 21707 550 32.2842 0.7579
SPT5_DMSO_Serum5 29941 5183 174 32.0865 1.1487
SPT5_dTAG_Serum5 27491 5339 163 34.4706 1.1789
SPT5_DMSO_Serum30 40127 8249 217 33.569 1.1487
SPT5_dTAG_Serum30 43116 9935 273 34.0166 1
pSPT5_Thr806P_DMSO_Serum0 308173 126015 1983 41.9428 0.7937
pSPT5_Thr806P_DMSO_Serum5 99482 36701 590 40.1875 1.2114
pSPT5_Thr806P_dTAG_Serum5 95869 24542 511 30.8381 1.1447
CDK9_DMSO_Serum0 452080 333921 2867 73.5347 1.0073
CDK9_DMSO_Serum5 245142 135489 1484 55.8562 1
CDK9_dTAG_Serum5 233371 155879 1476 66.6648 1.2941
CDK9_DMSO_Serum30 210504 96449 1178 46.8914 0.9441
CDK9_dTAG_Serum30 306263 165895 1616 54.6788 0.7841
CDK7_DMSO_Serum0 112910 44695 960 44.4832 1
CDK7_DMSO_Serum30 130406 56403 1068 47.1684 1
CDK7_dTAG_Serum30 156589 65781 1416 46.2314 1
MED1_DMSO_Serum0 561891 404285 5047 72.9469 0.9103
MED1_DMSO_Serum30 505410 358961 4949 71.6832 0.951
MED1_dTAG_Serum30 450515 316463 3977 70.6458 1
TFIIB_DMSO_Serum0 189012 148324 1204 77.4249 1
TFIIB_DMSO_Serum30 186992 116588 974 62.159 0.9086
TFIIB_dTAG_Serum30 115949 82997 689 70.4418 1.1447

Antibodies

HA/Millipore/05-905/ChIP (5 µg/ChIP)
RPB3/Millipore/ABE999/ChIP (5 µg/ChIP)
SPT5/Bethyl/Thermo/A300-868A/ChIP (5 µg/ChIP)
CDK9/Cell Signaling Technologies/2316/ChIP (18 µL/ChIP)
pS2 Pol II/Millipore/04-1571/ChIP (10 µg/ChIP)
pS5 Pol II/Active Motif/61085/ChIP (10 µg/ChIP)
TFIIB/Santa Cruz Biotechnologies/sc-271736/ChIP (5 µg/ChIP)
MED1/Bethyl/A300-793A/ChIP (5 µg/ChIP)
CDK7/Bethyl/A300-405A/ChIP (5 µg/ChIP)
pSPT5/gift_Robert_Fisher/ChIP (7 uL/ChIP)

Peak calling parameters

macs2 callpeak -t ${main}/MACS/HA/DS0_HA_merged-FS.bam -c ${main}/MACS/D_S0_Input-FS.bam -n HA_DMSO_SS0--outdir ${main}/MACS/HA -f BAMPE -g hs -q 0.01 -B --broad

Data quality

Raw fastq data files were always ran through fastqc/0.11.8 to ensure data quality, including duplication rate, presence of adapters, along with other quality statistics. Duplicates were always removed in ChIP-Seq data.

Software

Detailed scripts, tutorials, and input files needed to run all analysis for ChIP-Seq for this paper are under our lab GitLab page. Briefly, raw fastq data files were ran through fastqc/0.11.8 and low quality reads/adapter contaminations were removed using trimgalore/0.6.4. Reads were mapped to the hg38 human reference genome using bowtie2/2.4.2. Reads were also mapped to the dm6 Drosophila genome to extract reads originating from the spike-in chromatin for ChIPs that were normalized using spike-ins. Duplicates were marked and removed using picard/2.10.3 and then files were sorted and indexed using samtools/1.6 in preparation for bigWig generation. Normalized bigWig files were made from sorted bam files using the bamCoverage command using deepTools/2.3.594. The following ChIP-Seqs were normalized using Drosophila spike-ins: HA, SPT5, pSPT5, CDK9, CDK7, TFIIB, and MED1. The following ChIP-Seqs were normalized to read depth (counts per millions or CPM using deepTools): Pol II, Ser5P Pol II, Ser2P Pol II. For spike-in normalization, scaling factors (Supplementary Table 5) were defined using deepTools command multiBamSummary and then inputted into bamCoverage for Bigwig generation for normalization. Importantly, all spike-in normalized datasets were compared to read-depth normalized datasets to ensure quality and agreement. Ser5P Pol II and Ser2P Pol II ChIP-Seq data were normalized to Pol II ChIP-Seq data using deepTools command bamCompare. Detailed alignment statistics for both human genome reads and spike-in coverage are included in Supplementary Table 5. The Integrated Genome Viewer (IGV) was used for visualization.
